# Sleep duration, vital exhaustion, and odds of spontaneous preterm birth: a case–control study

**DOI:** 10.1186/1471-2393-14-337

**Published:** 2014-09-27

**Authors:** Sandhya Kajeepeta, Sixto E Sanchez, Bizu Gelaye, Chunfang Qiu, Yasmin V Barrios, Daniel A Enquobahrie, Michelle A Williams

**Affiliations:** Department of Epidemiology, Harvard School of Public Health, 677 Huntington Avenue, Kresge Building, Room 500, Boston, MA 02115 USA; Universidad Peruana de Ciencias Aplicadas, Lima, Peru; Asociación Civil PROESA, Lima, Peru; Center for Perinatal Studies, Swedish Medical Center, Seattle, WA USA; Department of Epidemiology, University of Washington School of Public Health, Seattle, WA USA

**Keywords:** Sleep duration, Exhaustion, Preterm birth, Pregnancy

## Abstract

**Background:**

Preterm birth is a leading cause of perinatal morbidity and mortality worldwide, resulting in a pressing need to identify risk factors leading to effective interventions. Limited evidence suggests potential relationships between maternal sleep or vital exhaustion and preterm birth, yet the literature is generally inconclusive.

**Methods:**

We examined the relationship between maternal sleep duration and vital exhaustion in the first six months of pregnancy and spontaneous (non-medically indicated) preterm birth among 479 Peruvian women who delivered a preterm singleton infant (<37 weeks gestation) and 480 term controls who delivered a singleton infant at term (≥37 weeks gestation). Maternal nightly sleep and reports of vital exhaustion were ascertained through in-person interviews. Spontaneous preterm birth cases were further categorized as those following either spontaneous preterm labor or preterm premature rupture of membranes. In addition, cases were categorized as very (<32 weeks), moderate (32–33 weeks), and late (34- <37 weeks) preterm birth for additional analyses. Logistic regression was used to estimate adjusted odds ratios (aORs) and 95% confidence intervals (CIs).

**Results:**

After adjusting for confounders, we found that short sleep duration (≤6 hours) was significantly associated with preterm birth (aOR = 1.56; 95% CI 1.11-2.19) compared to 7–8 hours of sleep. Vital exhaustion was also associated with increased odds of preterm birth (aOR = 2.41; 95% CI 1.79-3.23) compared to no exhaustion (*P*_trend_ <0.001). These associations remained significant for spontaneous preterm labor and preterm premature rupture of membranes. We also found evidence of joint effects of sleep duration and vital exhaustion on the odds of spontaneous preterm birth.

**Conclusions:**

The results of this case–control study suggest maternal sleep duration, particularly short sleep duration, and vital exhaustion may be risk factors for spontaneous preterm birth. These findings call for increased clinical attention to maternal sleep and the study of potential intervention strategies to improve sleep in early pregnancy with the aim of decreasing risk of preterm birth.

## Background

Preterm birth is a leading cause of perinatal morbidity and mortality worldwide, affecting 15 millions infants and constituting over one million deaths each year [1–3]. The etiology of preterm birth remains unclear, however, lifestyle factors such as drug and alcohol use, lack of physical activity, and psychosocial stress have been shown to be associated with increased risks of preterm birth [4]. Although additional factors may be stronger predictors of preterm birth such as previous preterm deliveries, periodontal disease, and maternal anemia [5, 6], lifestyle and behavioral factors are of particular interest due to their modifiability. Pregnant women are at an increased risk for sleep deprivation and exhaustion, yet very few studies have examined the relationship between maternal sleep and preterm birth [7–10]. Moreover, results from the few studies on this topic have been inconsistent due in part to variations in the types of sleep traits measured and when in pregnancy those traits were assessed. Recently, Micheli et al. [11] reported that maternal short sleep duration (≤5 hours per night) in the third trimester was associated with a 1.7-fold (95% confidence interval (CI) 1.1-2.8) increased risk of preterm birth. However, because maternal sleep was assessed in the third trimester, the association may be the result of sleep insufficiency attributable to symptoms of preterm labor. Other investigative teams have assessed risk of preterm birth in relation to maternal habitual sleep duration during early pregnancy. For example, the authors of a 2012 case–control study found no association between sleep duration (<7 hours or >8 hours), measured in the second trimester, and risk of preterm birth [12]. However, the authors were unable to distinguish between spontaneous and medically indicated preterm birth.

Apart from the small and inconsistent literature concerning sleep insufficiency and preterm delivery risk, a small emerging literature suggests that sleep disruption and other sleep traits, such as long sleep latency and poor sleep quality, may be associated with adverse perinatal outcomes, including preterm birth. For instance, Strange et al. [13] in their study of 220 pregnant women reported that preterm cases had a longer average sleep latency during the second trimester than term controls (26.09 vs. 18.53 minutes, respectively). More recently, Hernandez-Diaz and colleagues [14] reported that maternal complaints of sleep disturbance were more than twice as common in the 24-hour period before the onset of spontaneous preterm labor (SPTL) and preterm premature rupture of membrane (PPROM) compared to complaints during the control period of 48–76 hours prior to SPTL/PPROM (26% vs. 12% of participants). Findings from this case-crossover analysis identified sleep disturbance as an acute trigger of spontaneous preterm birth (odds ratio (OR) = 4.5; 95% CI 1.5-13.3). On balance, available evidence, albeit sparse, suggests that short sleep duration, poor sleep quality, and longer sleep latency may be novel modifiable risk factors for preterm delivery. However, given the inconsistency of findings, particularly findings concerning the relationship between short sleep duration and preterm birth, we completed a secondary data analysis of information from a large case–control study of pregnant Peruvian women who were asked to report habitual sleep duration and feelings of exhaustion during the first six months of pregnancy, with the objective of evaluating the associations between these exposures and preterm birth.

## Methods

### Study population and selection of cases and controls

A detailed description of the case–control study has been previously reported [15, 16]. Briefly, the study was conducted among women who delivered live births at the Hospital Nacional Dos de Mayo, the Instituto Nacional Materno Perinatal de Lima, and the Hospital Edgardo Rebagliati Martins in Lima, Peru, from January 2009 through July 2010. Cases were women with singleton pregnancies who had spontaneous (non-medically indicated) delivery before completing 37 weeks of gestation (22–36 weeks of gestation). Spontaneous preterm birth cases were identified by daily monitoring of all new deliveries at postpartum wards of participating hospitals. Women who delivered prior to 37 completed weeks of gestation as a result of medical intervention were not included in this study. Of the 515 eligible cases approached, 479 (93%) agreed to participate in the study. Controls were women who delivered a singleton infant at term (≥37 weeks of gestation) and were selected from the same hospital of delivery. An eligible control, delivering immediately after a case patient, was approached and recruited for the study. Of the 546 eligible controls approached, 480 (88%) agreed to participate in the study. Written informed consent was obtained from all participants. The ethical review boards of the Hospital Nacional Dos de Mayo, the Instituto Nacional Materno Perinatal de Lima, and the Hospital Edgardo Rebagliati Martins in Lima, Peru approved this study.

### Data collection and variable specification

Enrolled participants were asked to take part in a 45-minute in-person interview in which research-trained midwives used a standardized, structured Spanish-language questionnaire to elicit information regarding maternal socio-demographic characteristics, lifestyle habits, and medical and reproductive histories. Trained obstetricians reviewed participants’ labor and delivery medical records and prenatal medical records. Abstractions were completed using a standardized abstraction form. Information abstracted from medical records included participants’ medical and reproductive histories, pregnancy complications, condition of the newborn, and clinical details concerning labor and delivery characteristics.

Preterm birth was defined using American College of Obstetricians and Gynecologists (ACOG) guidelines [17]. Gestational age was based on the date of the last menstrual period and was confirmed by an ultrasound examination before 20 weeks. We categorized spontaneous preterm birth cases according to the two pathophysiological groups previously described (i.e., spontaneous preterm labor and delivery and preterm premature rupture of membranes) [18]. Spontaneous preterm labor and delivery (SPTL) cases were comprised of women whose medical records indicated an obstetrician diagnosis of spontaneous labor onset (with intact fetal membranes) and delivery prior to the completion of 37 weeks gestation. Preterm premature rupture of membranes (PPROM) cases were comprised of women whose medical records indicated an obstetrician diagnosis of rupture of fetal membranes (prior to the onset of labor) and delivery prior to the completion of 37 weeks gestation. Finally, we categorized preterm birth cases according to gestational age at delivery (i.e., very preterm birth, defined as delivery prior to the completion of <32 weeks gestation; moderate preterm birth, defined as delivery between 32 and 33 weeks gestation; and late preterm birth, defined as 34- <37 weeks gestation).

Information collected during the interviews included maternal age, marital status, employment status during pregnancy, medical history, and smoking and alcohol consumption during pregnancy. Maternal nightly sleep duration during the first 6 months of pregnancy was determined by the response to the question: “During the first 6 months of your pregnancy, how many hours per night did you sleep?” Responses were recorded as integers. Any amount of sleep that was less or more than 7–8 hours/night was indicative of poor sleep quality or abnormal sleep duration [12, 19]. Validation studies have reported that subjective measures of sleep duration and sleep efficiency are highly correlated with objective measures [20, 21], however others have found poor agreement between subjective and objective measures [22, 23]. Maternal report of vital exhaustion in early pregnancy was ascertained by asking women: “During the first 6 months of your pregnancy, how often did you feel exhausted (except after exercise)?” Response choices were: (1) never; (2) once per month; (3) 2–3 times per month; (4) 4 times per month; (5) every week; and (6) every day. For multivariable analyses, we collapsed responses into a dichotomous variable with “never” comprising the responses never, and “ever” comprising all other responses. We also created an ordinal variable with the categories: (1) Never, (2) 1–3 times per month; (3) 4 times per month or weekly; and (4) daily. We limited questions to early pregnancy because this period preceded any clinical manifestation of preterm labor or rupture of membrane. Other covariates considered in these analyses included maternal age and reproductive and medical histories. Also considered were maternal pre-pregnancy weight, educational attainment, annual household income, occupation, and cigarette smoking and alcohol consumption during pregnancy. Maternal age at the time of interview was expressed in years. Parity was reported as the number of previous pregnancies lasting more than 22 weeks gestation. Maternal educational attainment was based on self-reports.

#### Statistical analysis

We examined the frequency distribution of maternal socio-demographic characteristics and other covariates according to case and control status. To estimate the relative association between sleep duration and risk of preterm birth, logistic regression procedures were performed to calculate maximum likelihood estimates of ORs and 95% CIs that were adjusted for potential confounding [24]. We considered maternal educational attainment, pre-pregnancy weight, planned pregnancy, use of prenatal care services, employment status, cigarette smoking, alcohol consumption, and use of illicit drugs during pregnancy as possible confounders in these analyses. Body mass index was not included as a potential covariate because maternal height was not measured. Final logistic regression models included variables that altered the unadjusted OR by at least 10%, as well as those covariates of *a priori* interest (i.e., maternal age). Because a number of studies have suggested curvilinear relationships between sleep duration and pregnancy outcomes [25–27], we explored the possibility of a nonlinear relation between maternal sleep duration and preterm birth risk using the generalized additive modeling (GAM) procedure [28] by using S-PLUS (version 6.1, Insightful Corp 2002). Further, we examined potential effect modification by vital exhaustion by using stratified analyses and including multiplicative interaction terms in logistic regression models as previously described [24]. The same analysis procedure applied to examine the association between vital exhaustion and preterm delivery, including an examination of potential effect modification by sleep duration. Multinomial logistic regression models were designed to assess risk of sub-types of preterm birth (i.e., SPTL; PPROM; and very, moderate, and late preterm birth). All reported P-values are two-tailed. All analyses were performed using STATA 9.0 statistical software (Stata, College Station, USA). Prior to initiating the study, we estimated that a study size of 400 cases and an equal number of controls would be sufficient (>85% power) for estimating odds ratios of ≥2.0 if exposure frequencies were ≥10% and significance was set at 0.05.

## Results

The socio-demographic and reproductive characteristics as well as the infant outcomes of preterm cases and term controls are presented in Table 1. The two groups were similar with regard to most socio-demographic and reproductive characteristics with the exception of planned pregnancy, prenatal care, prenatal vitamin use, and pre-pregnancy weight. Preterm cases were less likely to identify the pregnancy as planned (31.7% vs. 43.5%), more likely to have received no prenatal care (15.7% vs. 4.0%), more likely to have taken no prenatal vitamins (24.8% vs. 14.2%), and had a lower average pre-pregnancy weight than did controls (56.7 kg vs. 58.0 kg). The cases and controls also differed by infant birth weight and the proportion of low birth weight infants (<2500 g), with cases having a lower average infant birth weight and a greater proportion of low birth weight infants than controls, as expected.

**Table 1:** **Socio-demographic and reproductive characteristics and infant outcomes in the study sample, Lima, Peru, 2009-2010**

	Spontaneous preterm birth				
	Term controls (N = 480)		Cases (N = 479)		
Characteristics	n	%	n	%	P-value
Maternal age at delivery (years)	28.3 ± 6.5		28.2 ± 6.6		0.74
<20	42	8.8	49	10.2	0.49
20-29	235	49.0	228	47.6	
30-34	94	19.6	107	22.3	
≥35	109	22.7	95	19.8	
Primiparity	199	41.5	205	42.8	0.68
≤High school education	335	69.8	319	66.6	0.29
Employed during pregnancy	195	40.6	177	37.0	0.24
Planned pregnancy	209	43.5	152	31.7	<0.001
No Prenatal care	19	4.0	75	15.7	<0.001
No Prenatal vitamin	68	14.2	119	24.8	<0.001
Smoked during pregnancy	1	0.2	4	0.8	0.22
Alcohol use during pregnancy	157	32.7	150	31.3	0.64
Illicit drug use during pregnancy	0	0.0	3	0.6	---
Pre-pregnancy weight (kg)	58.0 ± 9.8		56.7 ± 10.0		0.04
Infant birth weight (grams)	3393 ± 462		1999 ± 663		<0.001
Low birth weight infant (<2500grams)	14	2.9	381	76.5	<0.001

Continuous measures: Mean ± SD (SD = standard deviation).

**Figure 1:**
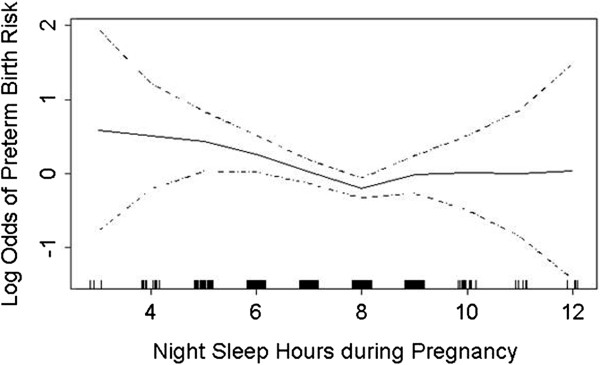
**Relationship between maternal sleep duration and odds of spontaneous preterm birth (solid line), with 95% confidence intervals (dotted lines) using the generalized additive model.**

The prevalence of short sleep duration (≤6 hours) was 22.3% among preterm cases and 16.5% among term controls (Table 2). The corresponding figures for long sleep duration (≥9 hours) were 18.4% for cases and 15.8% for controls. Table 2 presents the associations of maternal sleep duration and vital exhaustion during the first six months of pregnancy with preterm birth. We noted that the odds of preterm birth were increased 1.55-fold for women with short sleep duration (OR = 1.55; 95% CI 1.11-2.16) and 1.33-fold for women who reported long sleep duration (OR = 1.33; 95% CI 0.94-1.87) as compared with women who reported sleeping 7–8 hours per night. After adjusting for confounding by maternal age, pre-pregnancy weight, prenatal vitamin consumption, and unplanned pregnancy status, short sleep duration remained associated with increased odds of preterm birth, compared with sleep duration of 7–8 hours (adjusted odds ratio (aOR) = 1.56; 95% CI 1.11-2.19). The aOR for preterm birth with long sleep duration was 1.34 (95% CI 0.94-1.91). When we modeled the risk of preterm birth in relation to maternal sleep duration expressed as a continuous variable using a GAM, we noted an expected “U” shape relation between preterm birth risk and maternal sleep duration (Figure 1). The odds of preterm birth were increased by 2.41-fold among women who reported feeling a sense of vital exhaustion in early pregnancy (unrelated to exercise) (aOR = 2.41; 95% CI 1.79-3.23) compared to women who did not report any symptoms (Table 2). Additionally, we found a statistically significant linear trend between frequency of complaint of vital exhaustion and odds of spontaneous preterm birth (*P*_trend_ <0.001). Though, weekly complaints of vital exhaustion were associated with greater odds of preterm birth than were daily complaints. These associations remained statistically significant and were of similar magnitude when both sleep duration and vital exhaustion were included in the same logistic regression model.

**Table 2:** **Odds Ratio (OR) and 95% confidence interval (CI) of spontaneous preterm birth according to maternal sleep duration and vital exhaustion during the first six months of pregnancy, Lima, Peru, 2009-2010**

	Term Controls (N = 480)		Cases (N = 479)		Unadjusted		Adjusted*		Adjusted**	
Exposure parameters	n	%	n	%	OR	(95% CI)	OR	(95% CI)	OR	(95% CI)
**Hours of sleep per night**										
≤ 6 hours	79	16.5	107	22.3	**1.55**	**(1.11-2.16)**	**1.56**	**(1.11-2.19)**	**1.53**	**(1.08-2.17)**
7-8 hours	325	67.7	284	59.3	1.00	referent	1.00	referent	1.00	referent
≥ 9 hours	76	15.8	88	18.4	1.33	(0.94-1.87)	1.34	(0.94-1.91)	**1.50**	**(1.04-2.16)**
**Complaint of vital exhaustion**										
Never	185	38.5	99	20.7	1.00	referent	1.00	referent	1.00	referent
Ever	295	61.5	380	79.3	**2.41**	**(1.81-3.21)**	**2.41**	**(1.79-3.23)**	**2.46**	**(1.83-3.32)**
*1-3 times/Month*	*204*	*42.5*	*201*	*41.9*	***1.84***	***(1.35-2.52)***	***1.89***	***(1.38-2.60)***	***1.97***	***(1.43-2.72)***
*4 times/Month - Weekly*	*73*	*15.2*	*156*	*32.6*	***3.99***	***(2.76-5.78)***	***3.85***	***(2.64-5.62)***	***3.91***	***(2.67-5.73)***
*Daily*	*18*	*3.8*	*23*	*4.8*	***2.39***	***(1.23-4.64)***	***2.42***	***(1.23-4.76)***	***2.23***	***(1.12-4.45)***
*P-value for trend*						<0.001		<0.001		<0.001

*Adjusted for maternal age, pre-pregnancy weight, unplanned pregnancy, and no vitamin use during pregnancy.

**Same as above but now also include sleep duration and vital exhaustion in the model.

Bolded estimates are statistically significant at the α = 0.05 level.

The aORs of preterm subtypes, spontaneous preterm labor (SPTL) and preterm premature rupture of membranes (PPROM), according to maternal sleep duration and complaint of vital exhaustion, are shown in Table 3. The odds of SPTL and of PPROM increased by 1.51-fold and 1.60-fold, respectively, among women who reported short sleep duration compared with women who reported sleep duration of 7–8 hours. For women who reported long sleep duration, the odds of SPTL and of PPROM were 1.50 and 1.17 times greater than the odds among women who reported sleep duration of 7–8 hours. Women who complained about feelings of vital exhaustion had 2.21-fold higher odds (aOR 2.21; 95% CI 1.54–3.15) of experiencing SPTL and 2.66-fold higher odds (aOR 2.66; 95% CI 1.81-3.89) of experiencing PPROM compared with women who did not complain of vital exhaustion during early pregnancy. The odds of SPTL and PPROM increased as frequency of complaint of vital exhaustion increased (both *P*_trend_ <0.001).

**Table 3:** **Odds Ratio (OR) and 95% Confidence Interval (CI) of spontaneous preterm birth sub-types according to maternal sleep duration and vital exhaustion during the first six months of pregnancy, Lima, Peru, 2009-2010**

	Term controls (N = 480)	Spontaneous Preterm Labor (SPTL) (N = 245)		Preterm Premature Rupture of Membrane (PPROM) (N = 234)	
Exposure parameters	n	n	OR* (95% CI)	n	OR* (95% CI)
**Hours of sleep per night**					
≤ 6 hours	79	51	**1.51 (1.00-2.29)**	56	**1.60 (1.06-2.40)**
7-8 hours	325	143	1.00 (referent)	141	1.00 (referent)
≥ 9 hours	76	51	1.50 (0.99-2.27)	37	1.17 (0.74-1.83)
**Complaint of vital exhaustion**					
Never	185	55	1.00 (referent)	44	1.00 (referent)
Ever	295	190	**2.21 (1.54-3.15)**	190	**2.66 (1.81-3.89**
*1-3 times/Month*	*204*	*106*	***1.81 (1.23-2.67)***	*95*	***2.00 (1.32-3.02)***
*4 times/Month - Weekly*	*73*	*73*	***3.34 (2.13-5.24)***	*83*	***4.47 (2.81-7.10)***
*Daily*	*18*	*11*	*2.17 (0.95-4.93)*	*12*	***2.73 (1.21-6.16)***
*P-value for trend*			<0.001		<0.001

*Adjusted for maternal age, pre-pregnancy weight, unplanned pregnancy, and no vitamin use during pregnancy.

Bolded estimates are statistically significant at the α = 0.05 level.

The aORs of very preterm birth (<32 weeks), moderate preterm birth (32–33 weeks), and late preterm birth (34– < 37 weeks) in relation to maternal sleep duration and complaint of vital exhaustion are shown in Table 4. Women who reported long sleep duration had increased odds of moderate preterm birth, compared with the referent group (aOR 1.98; 95% CI 1.08–3.65). Those women who reported short sleep duration had increased odds of late preterm birth as compared with the referent group (aOR 1.64; 95% CI 1.11-2.43). Vital exhaustion was associated with increased odds of early (aOR 2.40; 95% CI 1.51-3.82), moderate (aOR 1.96; 95% CI 1.13-3.41) and late (aOR 2.58; 95% CI 1.80-3.69) preterm birth. Among those who reported vital exhaustion, the odds of each preterm birth sub-group increased with increasing frequency of complaint of vital exhaustion (all *P*_trend_ ≤0.004).

**Table 4:** **Odds Ratio (OR) and 95% Confidence Interval (CI) of spontaneous preterm birth severity according to maternal sleep duration and vital exhaustion during the first six months of pregnancy, Lima, Peru, 2009-2010**

	Term controls (N = 480)	Very preterm < 32 weeks (N = 135)		Moderate preterm 32–33 weeks (N = 78)		Late preterm 34- < 37 weeks (N = 266)	
Exposure parameters	n	n	OR* (95% CI)	n	OR* (95% CI)	n	OR* (95% CI)
**Hours of sleep per night**							
≤ 6 hours	79	25	1.25 (0.74-2.11)	18	1.82 (0.98-3.37)	64	**1.64 (1.11-2.43)**
7-8 hours	325	82	1.00 (referent)	41	1.00 (referent)	161	1.00 (referent)
≥ 9 hours	76	28	1.48 (0.89-2.47)	19	**1.98 (1.08-3.65)**	41	1.10 (0.72-1.70)
**Complaint of vital exhaustion**							
Never	185	28	1.00 (referent)	19	1.00 (referent)	52	1.00 (referent)
Ever	295	107	**2.40 (1.51-3.82)**	59	**1.96 (1.13-3.41)**	214	**2.58 (1.80-3.69)**
*1-3 times/Month*	*204*	*66*	***2.22 (1.36-3.63)***	*34*	*1.65 (0.91-3.00)*	*101*	***1.81 (1.22-2.69)***
*4 times/Month - Weekly*	*73*	*36*	***3.07 (1.73-5.45)***	*20*	***2.68 (1.34-5.35)***	*100*	***4.70 (3.03-7.30)***
*Daily*	*18*	*5*	*1.90 (0.64-5.60)*	*5*	*2.74 (0.90-8.31)*	*13*	***2.59 (1.18-5.72)***
*P-value for trend*			<0.001		0.004		<0.001

*Adjusted for maternal age, pre-pregnancy weight, unplanned pregnancy, no vitamin use during pregnancy.

Bolded estimates are statistically significant at the α = 0.05 level.

Finally, we completed an exploratory analysis to assess the odds of preterm birth in relation to independent and joint exposure to short/long sleep duration and any complaint of vital exhaustion in early pregnancy. As shown in Table 5, we observed some evidence suggestive of effect modification though the P-values for interaction terms were not statistically significant, likely due to insufficient statistical power for effect modification analyses. Women who reported short sleep duration and complained of exhaustion had 3.58-fold increased odds of preterm birth (aOR = 3.58; 95% CI 2.21-5.78) as compared with women who reported sleeping 7–8 hours and had no complaints of exhaustion (P-value for this multiplicative interaction term = 0.60). Women who reported sleeping long durations and complained of vital exhaustion had 3.74-fold increased odds of preterm birth (aOR = 3.74; 95% CI 2.20-6.35) as compared with the referent group (P-value for this multiplicative interaction term = 0.40). The results from these analyses also suggest that vital exhaustion may be more strongly associated with preterm birth than sleep duration. However, as noted earlier, inferences from these exploratory analyses are limited by the relatively small sample size and low power to formally test for statistical interactions.

**Table 5:** **Odds Ratio (OR) and 95% Confidence Interval (CI) of spontaneous preterm birth for joint categories of maternal sleep duration and complaints of vital exhaustion during the first six months of pregnancy, Lima, Peru, 2009-2010**

	Term controls (N = 480)		All spontaneous preterm birth (N = 479)		Unadjusted OR		Adjusted OR*	
Night hours of sleep & exhaustion	n	%	n	%	OR	(95% CI)	OR	(95% CI)
7-8 hours & no exhaustion	117	24.4	57	11.9	1.00	Referent	1.00	Referent
≤ 6 hours & no exhaustion	27	5.6	16	3.3	1.22	(0.61-2.44)	1.30	(0.64-2.63)
≥ 9 hours & no exhaustion	41	8.5	26	5.4	1.30	(0.73-2.34)	1.22	(0.67-2.22)
7-8 hours & any exhaustion	208	43.3	227	47.4	**2.24**	**(1.55-3.24)**	**2.22**	**(1.52-3.23)**
≤ 6 hours & any exhaustion	52	10.8	91	19.0	**3.59**	**(2.26-5.72)**	**3.58**	**(2.21-5.78)**
≥ 9 hours & any exhaustion	35	7.3	62	12.9	**3.64**	**(2.16-6.13)**	**3.74**	**(2.20-6.35)**
Interaction p-value (short sleep & any exhaustion)					0.50		0.60	
Interaction p-value (long sleep & any exhaustion)					0.56		0.40	

*Adjusted for maternal age, pre-pregnancy weight, unplanned pregnancy, no vitamin use during pregnancy.

Bolded estimates are statistically significant at the α = 0.05 level.

## Discussion

In our study population, 36.5% of participants reported either short or long sleep duration and over 70% of participants reported vital exhaustion, supporting the evidence that sleep deprivation and sleep disturbance during pregnancy is highly prevalent [7–10]. Our results suggest that maternal sleep duration and vital exhaustion are statistically significantly associated with preterm birth. Short sleep duration (≤6 hours of nightly sleep), long sleep duration (≥9 hours of nightly sleep), and any complaint of vital exhaustion were associated with increased odds of all subtypes of spontaneous preterm birth (SPTL; PPROM; very, moderate, and late preterm birth). Additionally, we found some evidence that the effects of sleep duration and vital exhaustion on preterm birth may increase when considered jointly.

These results are largely consistent with some [11, 29, 30], though not all [12, 13], prior studies that have assessed the relationship between sleep duration and preterm birth. Klebanoff et al. [29] found that pregnant medical residents who worked ≥100 hours per week during pregnancy had a greater risk of preterm birth than pregnant residents who worked <100 hours per week and Osborn et al. [30] found similar results when comparing pregnant medical residents to the pregnant wives of male medical residents. Although sleep duration was not directly measured in these studies, the findings suggest that sleep deprivation is associated with a greater risk of preterm birth and our results provide more evidence of this association. More recently, Micheli et al. [11] identified a statistically significant association between short sleep duration (≤5 hours) in late pregnancy and an increased risk of preterm birth. Our results reinforce these findings and further suggest that this association is not simply due to a manifestation of preterm labor symptoms in late pregnancy. The associations we identified between short and long sleep duration and increased odds of preterm birth contrast with the results of Guendelman et al. [12] and Strange et al. [13], who found no association between sleep duration (either short or long) and odds of preterm delivery. Our findings may differ from those of Guendelman et al. [12] and Strange et al. [13] for several reasons. In the present study, sleep duration was reported for the first six months of pregnancy, whereas others [12, 13] measured sleep duration only in the second trimester of pregnancy. By including sleep data from the first trimester of pregnancy in our analysis, we can be more certain that changes in sleep are not attributable to early symptoms of preterm labor or premature rupture of membranes. Another plausible reason for the inconsistent findings is that the authors did not differentiate between spontaneous and medically indicated preterm birth. For our present analysis, we focused on assessment of risk factors for spontaneous preterm birth. Finally, our study was conducted among Peruvian women who delivered in hospitals in Lima and the results may not be generalizable to other maternal populations.

Previous studies have identified poor sleep quality and longer sleep latency as potential risk factors for preterm birth, yet ours is the first study, to our knowledge, to evaluate the association between vital exhaustion in the first six months of pregnancy and preterm birth [13, 14]. Our results indicate that vital exhaustion is associated with approximately a 2-fold increase or greater in the odds of all subtypes of spontaneous preterm birth suggesting that vital exhaustion may be a novel risk factor for preterm birth. This contributes new evidence to the limited data on the influences of sleep quality, represented, at least in part, by chronic vital exhaustion in early pregnancy, on preterm birth. Additionally, our exploratory analysis of the joint effects of sleep duration and vital exhaustion on preterm birth suggest that vital exhaustion may be more strongly associated with preterm birth. This is clinically plausible because alterations in sleep duration may represent normal physiological changes attributable to pregnancy while exhaustion may be a stronger indicator of sleep insufficiency resulting in compromised daytime functioning. Studies that include measures of sleep duration, sleep quality and circadian misalignment are needed to more thoroughly assess this hypothesis.

There are multiple potential mechanisms through which maternal sleep deprivation and preterm birth may be associated. For example, research suggests that inflammatory cytokines may play a major role in the initiation of preterm labor [8, 31, 32]. Studies have identified higher levels of inflammatory cytokines, including interleukin-6 (IL-6) and interleukin-8 (IL-8), in women who had delivered prematurely compared to women who had not [31, 32]. Increased concentrations of IL-6 and IL-8 have also been found in pregnant women who report short sleep duration or poor sleep efficiency [33]. The associations between sleep deprivation and increased inflammatory cytokine concentrations and between inflammatory cytokines and preterm birth demonstrate a plausible mechanism for the observed associations.

Another potential mechanism for the observed associations of sleep deprivation, vital exhaustion and preterm birth involves the hypothalamic-pituitary-adrenal (HPA) axis, the hormonal system that appears to be most strongly associated with preterm labor [34, 35]. There is evidence of an association between sleep disturbances and the HPA axis in the general population [36–38]. Thus, a plausible pathway for the association between maternal sleep and risk of preterm birth is through alterations in the HPA axis secondary to sleep disturbances. However, more research is needed to thoroughly explore relationships between maternal early pregnancy sleep habits, sleep disturbances, HPA axis activation and perinatal outcomes, including preterm labor and premature rupture of membranes. Finally, obstructive sleep apnea and sleep-disordered breathing, symptoms of which include fatigue and excessive daytime sleepiness [39], are associated with multiple pregnancy outcomes [40, 41]. Such sleep disorders may play a mediating role in the association between sleep duration and exhaustion and preterm birth, though the association between sleep apnea and preterm birth has not been fully explored.

The present study has several strengths including the large numbers of preterm cases and controls as well as our relatively high participation rates (93% for cases and 88% for controls). Our confirmation of gestational age data via ultrasound examination and use of obstetrician diagnoses to define SPTL and PPROM help to mitigate concerns of misclassification. Additionally, we used sleep data from the first six months of pregnancy to help ensure that sleep changes were not the result of preterm labor symptoms. However, there are multiple limitations that should be acknowledged when interpreting these results. First, reports of maternal nightly sleep duration were categorized as integer values in order to minimize recall error. Consequently, our results require an assumption of constant effect within integer categories. Second, sleep duration and experience of vital exhaustion were obtained through retrospective self-report, so there is a possibility of recall bias. Yet, as previously mentioned, our results are generally consistent with those of Micheli et al. [11], which are not subject to recall bias because maternal sleep data was collected prior to birth. Additionally, because the accuracy of subjective sleep measures is ambiguous [20–23], it may be beneficial for future studies to use objective measures of sleep, such as wrist actigraphy, in combination with self-report to measure both perceived and actual sleep patterns. Finally, although we believe limiting our exposure period to the first six months of pregnancy is a strength of our study, we recognize that a strong design for future studies of maternal habitual sleep duration and pregnancy outcome should include serial assessments that more thoroughly describe sleep measures across the entire pregnancy.

## Conclusions

In conclusion, the present study identifies short and long maternal sleep duration and vital exhaustion in the first six months of pregnancy as potential modifiable risk factors for preterm birth. Many pregnant women are affected by sleep deprivation and disorders; thus, this knowledge could significantly improve birth outcomes worldwide [7–10]. There have been multiple intervention studies aimed at improving sleep in pregnancy and the early post-partum period with the goal of decreasing rates of post-partum depression [42–44]. Additionally, Blyton et al. [45] used positive airway pressure to treat sleep disordered breathing during pregnancy and was able to increase the average number of fetal movements in women with preeclampsia from 319 to 592 per night (P <0.0001). On balance, our findings coupled with those from an emerging, though small, experimental literature [42–45], suggest that expanded investment in research focused on assessing pregnancy outcomes among women treated for sleep disorders during pregnancy studies is warranted. Increased health education on the importance of maternal sleep health hygiene and increased clinical awareness of the influences of sleep deprivation during pregnancy on maternal and child health may also help alleviate the global burden of preterm birth.

## Abbreviations

aOR: Adjusted odds ratio
CI: Confidence interval
SPTL: Spontaneous preterm labor
PPROM: Preterm premature rupture of membrane
OR: Odds ratio
ACOG: American College of Obstetricians and Gynecologists
GAM: Generalized additive modeling
IL-6: Interleukin-6
IL-8: Interleukin-8
HPA: Hypothalamic-pituitary-adrenal
NIH: National Institutes of Health.
